# Cancer Informatics in the U.K.: The NCRI Informatics Initiative

**Published:** 2007-02-26

**Authors:** Fiona Reddington, J. Max Wilkinson, Robin Clark, Helen Parkinson, Peter Kerr, Richard Begent

**Affiliations:** 1 NCRI Informatics Initiative, London, U.K; 2 Formerly of the NCRI Informatics Initiative, now EBI, Hinxton, U.K; 3 Formerly of the NCRI Informatics Initiative, now Almac Diagnostics, Craigavon, U.K

**Keywords:** Data sharing, information integration, infrastructure, multi-disciplinary

## Abstract

The arrival of high-throughput technologies in cancer science and medicine has made the possibility for knowledge generation greater than ever before. However, this has brought with it real challenges as researchers struggle to analyse the avalanche of information available to them. A unique U.K.-based initiative has been established to promote data sharing in cancer science and medicine and to address the technical and cultural issues needed to support this.

The more we learn about cancer the more its complexity is revealed. Steady advances are being made in improving survival but it is increasingly clear that further progress depends significantly on coordinating research and on maximising its impact by sharing and integrating the vast amounts of data being generated. In the U.K., the National Cancer Research Institute (NCRI) was formally established as a key element of the National Cancer Plan in April 2001. For the first time the major cancer research funding bodies from the government, charity and private sectors have come together to form a partnership with the purpose of accelerating and advancing cancer research for the benefit of patients and the U.K. cancer research community. In 2002, the NCRI Board identified cancer informatics as an area of focus and the NCRI Informatics Initiative was established. The mission is simply stated but harder to fulfil: to use informatics to maximise the impact of cancer research.

A guiding principle for the NCRI Informatics Initiative is that the people best placed to advise on the future vision for informatics are those from the stakeholder communities, namely scientists, clinicians, bioinformaticists, computer scientists and patients. A “bottom-up” approach has been adopted which aims to address complex problems via the adoption of an agreed framework, authored substantially by the community themselves. A Task Force, comprising representatives from these stakeholder communities, has been established and provides a unique multi-disciplinary forum at which ideas are exchanged and collaborations are promoted. This Task Force has concluded that the U.K. vision for the Initiative should be for an internationally compatible informatics platform that facilitates access to data generated from research funded by NCRI Partner organisations, across the spectrum from genomics to clinical trials and population studies. The platform could also be used as a route to capture results, enabling the body of collated information to grow without adding to the burden of researchers. The vision is illustrated in Figure 1.

Whilst this is a laudable aim, the advent of high-throughput technologies has meant that, more than ever before, researchers are being faced with an avalanche of data which needs rigour and discipline in it’s collection and management if it is to be exploited to it’s full potential and translated into knowledge. A key premise of the NCRI Informatics Initiative is that data sharing should become the norm in cancer science and medicine, and that common data standards should be adopted to enable this to happen. Even in research fields where local or national repositories do exist, there is often a lack of interoperability between such resources that makes it difficult to exchange and interpret data in a meaningful fashion. As scientific research moves towards a systems biology approach, whereby the integration of data types from different disciplines is key, the need for “joined-up thinking” between disciplines is becoming critical. One way which the NCRI Informatics Initiative has engaged with the community on this issue is via hosting a series of workshops on key issues regarding data sharing between disciplines (e.g. Data Sharing in Clinical Trials, Integrating Functional Genomics and Clinical Trials and Human Genetic Variation). These workshops provide a unique opportunity for multi-disciplinary discussion, collating a community perspective on key issues via workshop reports, and publicising these findings via the NCRI Informatics website.

Many scientific communities, such as the functional genomics and clinical trials communities, have already realized the value of defined data sets and formats and have developed resources which their communities can use (e.g. MIAME, MAGE-OM, CONSORT). Other communities are following suit and the NCRI Informatics Initiative is strongly supportive of this approach and applauds the efforts of these respective groups. For it’s part, the Initiative has worked with the NCRI Partners to develop a Data Sharing Policy that encourages researchers to think about future usage of their data at an early stage and to adopt the use of standards for data collection and exchange where appropriate. To support this work, an online planning matrix has been developed which contains information about tools, standards and resources which researchers may find helpful in preparing a data sharing strategy (Figure 2).

It is key that high quality existing resources are leveraged wherever possible and the matrix encourages this by including information about international resources such as those from the cancer Biomedical Informatics Grid (caBIG^TM^) program in the U.S. and the European Bioinformatics Institute. Indeed, strategic partnerships with key international initiatives and projects will be crucial and data sharing needs to happen at this level too. Sharing information about lessons learnt, and success stories will ensure we do not repeat each other’s mistakes and move forward in the most productive way possible. This interaction has already started and representatives from both the National Cancer Institute (NCI) and EBI are members of the U.K. Task Force and tools and resources from these international partners are included in the NCRI Planning Matrix. Discussions are underway about the most appropriate way to work with these international resources in such a way that our respective differences are recognised and appreciated. This will range from raising awareness of each others work (via reciprocal links on websites) to organising and participating in joint events where appropriate.

Whilst the structure of the NCRI Informatics Initiative is different to that of the caBIG^TM^ program, there has been a real and sustained commitment from both groups to work together where possible. Whilst the U.S. has the advantage of a centralised team and large-scale financial resources, the U.K. benefits from well-established networks in the cancer community. The U.K. also has the potential to link with high-quality health-care data via the Secondary Uses Service (SUS) of the English National Health Service via its Connecting for Health (CfH) information system. The strategic alliance which exists between the caBIG program and NCRI Informatics is based on re-using each other’s resources where appropriate and benefiting from each other’s expertise. Indeed, the U.K. CancerGRID project is already working closely with the caBIG^TM^ program in several important areas including the NCICB Vocabulary and Common Data Elements workspace, the BRIDG clinical trial modelling project, the LexGrid project for the delivery of ontology and controlled vocabulary services, and the creation of four U.K. nodes on the NCI Center for Bioinformatics Grid.

Widening participation in the Initiative in the U.K. and internationally is a priority going forward. The NCRI Informatics Task Force consists of experts in research domains in biomedical informatics and provides two-way communication with the research community. It is also a conduit for the community to bring forward new ways in which informatics can be applied for benefit in cancer research. The Task Force generates formal and informal links in the research community. Separately, more formal links are also being forged with key stakeholders such as standards organisations, the U.K. e-Science Programme, journals and CfH and it’s equivalents across the individual countries that comprise the U.K. Within NCRI, we recognize that this is a problem that we can not solve alone, neither would we wish to try. We welcome the participation and collaboration of other Initiatives and groups. The U.K. cancer community has already demonstrated it’s willing participation in and benefits of, collaborative working environments through the success of the Network Organisations that have been established (e.g. National Cancer Research Network (NCRN)). However, the potential benefits of informatics are far-reaching and the ability to integrate with, and apply solutions to, other disease areas remains an important driver. The formation of the U.K. Clinical Research Collaboration (UKCRC) provides a unique forum for providing these links, and for facilitating interaction with CfH which also covers all aspects of healthcare.

The development of any ‘technical’ solutions to support data sharing, exchange and integration will need to be supported by appropriate training and financial support. Enthusiasm for the application of informatics is increasing and the scientists and clinicians of the future will need to be more versatile in their use of technologies, be able to work in multi-disciplinary teams to better understand complicated and often multi-dimensional aspects of their data, and be better prepared to translate discoveries into public benefit. The NCRI Informatics Coordination Unit has undertaken a training review. This review was approached from the perspective of culture change and the mechanisms that were in place to deliver this change in the U.K. The training activity in the U.K. is dispersed, multi-faceted and not generally confined to cancer research. Thus, training in this context is more about how to balance what could be achieved between the technical needs of individuals, the novel ways that cancer research is approached, and how this may be supported. Effective training in an integrated domain can only be effective when trainees understand the utility of multidiscipline models and can understand concepts from other domains.

The Informatics Initiative has established a case for investment in informatics via a quantified Business Case. An implementation plan, to include details of a peer-reviewed informatics funding stream, is currently being prepared to drive us forward and will provide resources to support the direct engagement of the community with the Initiative. We are aware that the challenges we face are not just technical and that significant further cultural change will need to take place before data sharing and informatics become routine in cancer science and medicine. To support this, the NCRI Informatics Coordination Unit is undertaking a proactive communication strategy via it’s website, www.cancerinformatics.org.uk, newsletters, participation in conferences and organisation of workshops and other fora in key areas. Furthermore, members of the Task Force have undertaken two high profile demonstration projects which illustrate generic applications and/or provide key infrastructure and thus demonstrate the benefit of informatics. They are:

## Imaging and Pathology

This demonstrator comes from radiological, microscopy, clinical trials, computer science and “integrative biology” communities. It is developing a framework which re-uses and adapts systems developed for imaging of breast cancer and applies them in rectal cancer, integrating magnetic resonance imaging (MRI) information with macroscopic data, microscopy and data from a clinical trial (Pitt-Francis et al. 2006; Slaymaker et al. 2006)

## The Platform Reference Model

The Platform Reference Model will provide a shared basis for understanding the key components of the information sharing and services platform envisaged by the NCRI Task Force. It will also provide a coherent basis for bringing together existing data sharing schemes. Development of Use Cases will direct how the platform is to be used and how it will deliver value to researchers and clinicians (Perrone et al. 2006; Begent el al. 2005).

In conclusion, a lot of work remains to be done before informatics can truly claim to be maximising the impact of cancer research. Resources will need to be provided, increased rigour will need to be applied to the collection of data, and large-scale training will have to be undertaken to underpin new technologies and working practices. However, the undeniable effort that will be required pales in comparison to the prize that is within our grasp, a shared international platform for cancer research that enables easy (but controlled) access to cancer related data for researchers and clinicians and improved outcomes for patients.

## Related web pages

National Cancer Research Institute: http://www.ncri.org.uk/

NCRI Informatics Initiative: http://www.cancerinformatics.org.uk/

NCRI Data Sharing Policy: http://www.cancerinformatics.org.uk/documents.htm#datasharing

NCRI Informatics Workshops: http://www.cancerinformatics.org.uk/workshops.htm caBIG: https://cabig.nci.nih.gov/

EBI: http://www.ebi.ac.uk/

CfH: http://www.connectingforhealth.nhs.uk/

CancerGRID: http://www.cancergrid.org

NCICB Vocabulary and Common Data Elements workspace: https://cabig.nci.nih.gov/workspaces/VCDE/

The BRIDG clinical trial modelling project: http://www.bridgproject.org/

The LexGrid project: http://informatics.mayo.edu/LexGrid/index.php?page=

The UK e-Science Programme: http://www.rcuk.ac.uk/escience/

National Cancer Research Network: http://www.ncrn.org.uk/

UK Clinical Research Collaboration: http://www.ukcrc.org/

NCRI Informatics Training Review: http://www.cancerinformatics.org.uk/documents.htm#TR

NCRI Informatics Business Case: http://www.cancerinformatics.org.uk/documents.htm#buscase

Imaging and Pathology project: http://www.cancerinformatics.org.uk/demo_projects.htm#imagpath

Platform Reference Model project: http://www.cancerinformatics.org.uk/demo_projects.htm#conmod

## Figures and Tables

**Figure 1:**
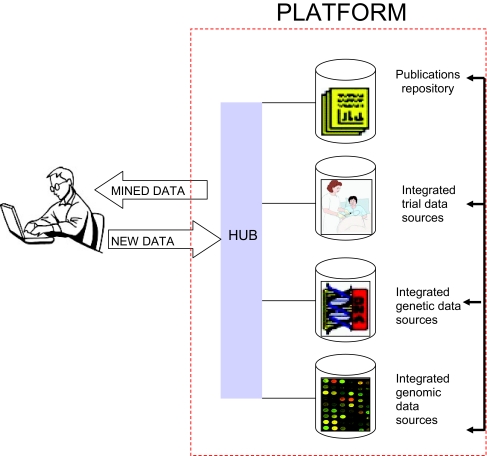
Schematic of the utility of a platform in assisting a cancer researcher.

**Figure 2:**
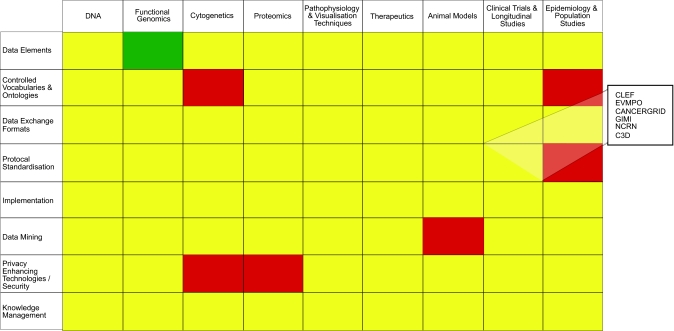
Schematic of NCRI Planning Matrix which contains details about informatics tools, project and resources.
